# Anti-Allergic Compounds from the Deep-Sea-Derived Actinomycete *Nesterenkonia flava* MCCC 1K00610

**DOI:** 10.3390/md15030071

**Published:** 2017-03-14

**Authors:** Chun-Lan Xie, Qingmei Liu, Jin-Mei Xia, Yuanyuan Gao, Quan Yang, Zong-Ze Shao, Guangming Liu, Xian-Wen Yang

**Affiliations:** 1 State Key Laboratory Breeding Base of Marine Genetic Resources, Key Laboratory of Marine Genetic Resources, Fujian Key Laboratory of Marine Genetic Resources, Third Institute of Oceanography, State Oceanic Administration, 184 Daxue Road, Xiamen 361005, China; xiechunlanxx@163.com (C.-L.X.); xiajinmei@tio.org.cn (J.-M.X.); shaozongze@tio.org.cn (Z.-Z.S.); 2 Department of Traditional Chinese Medicine, Guangdong Pharmaceutical University, Guangzhou 510006, China; yangquan7208@vip.163.com; 3 College of Food and Biological Engineering, Jimei University, 43 Yindou Road, Xiamen 36102, China; liuqingmei1229@163.com (Q.L.); yuanyuan201736@163.com (Y.G.); gmliu@jmu.edu.cn (G.L.)

**Keywords:** actinomycetes, *Nesterenkonia*, deep-sea, food allergy

## Abstract

A novel cyclic ether, nesterenkoniane (**1**), was isolated from the deep-sea-derived actinomycete *Nesterenkonia flava* MCCC 1K00610, together with 12 known compounds, including two macrolides (**2**, **3**), two diketopiperazines (**4**, **5**), two nucleosides (**6**, **7**), two indoles (**8**, **9**), three phenolics (**10**–**12**), and one butanol derivate (**13**). Their structures were established mainly on detailed analysis of the NMR and MS spectroscopic data. All 13 compounds were tested for anti-allergic activities using immunoglobulin E (IgE) mediated rat mast RBL-2H3 cell model. Under the concentration of 20 μg/mL, **1** exhibited moderate anti-allergic activity with inhibition rate of 9.86%, compared to that of 37.41% of the positive control, loratadine. While cyclo(d)-Pro-(d)-Leu (**4**) and indol-3-carbaldehyde (**8**) showed the most potent effects with the IC_50_ values of 69.95 and 57.12 μg/mL, respectively, which was comparable to that of loratadine (IC_50_ = 35.01 μg/mL). To the best of our knowledge, it is the first report on secondary metabolites from the genus of *Nesterenkonia*.

## 1. Introduction

**Figure 1:**
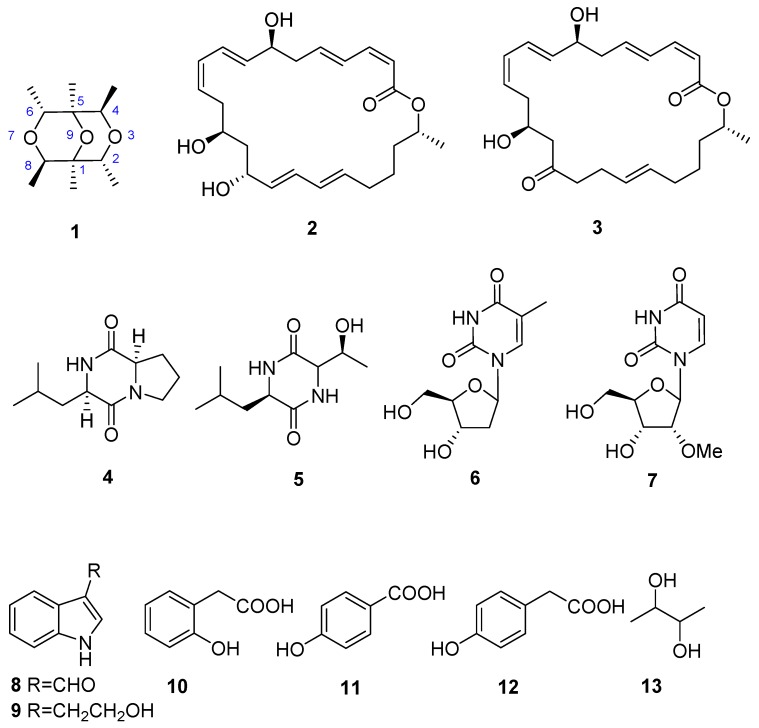
Compounds of marine actinomycete *Nesterenkonia flava* MCCC 1K00610.

Deep-sea organisms live in extreme conditions with no or little light, low concentration of oxygen, and intensely high pressure, necessitating a diverse array of biochemical and physiological adaptations that are essential for survival [1]. Marine microorganisms, particularly marine actinomycetes, have been proven to be rich sources of structurally novel and biologically active secondary metabolites with great pharmaceutical potential [2,3,4]. In fact, more than 60% of the clinical small molecule drugs have been obtained from natural products or their derivatives [5]. Actinomycetes have made extensive contributions to drug discovery [6]. It is estimated that around 75% of all known antibiotics are derived from secondary metabolites produced by filamentous actinomycetes [7]. These compounds display a wide range of biological activities, including antibacterial, antifungal, antiparasitic, antimalarial, immunomodulatory, anti-inflammatory, antioxidant, and anticancer activities. These diverse bioactive compounds include polyketides, alkaloids, fatty acids, peptides, and terpenes [8,9]. In our current research for bioactive compounds from deep-sea-derived microorganisms, the crude extract of *Nesterenkonia flava* MCCC 1K00610 showed potent anti-allergic activity on immunoglobulin E (IgE) mediated rat mast RBL-2H3 cells. Therefore, a systematic investigation on its chemical constituents was performed, which led to the isolation of one new and 12 known compounds (Figure 1). Herein, we report the fermentation, isolation, identification, and anti-allergic activity of these components.

## 2. Results

### 2.1. Structure Elucidation

The marine actinomycete MCCC 1K00610 was cultured on 18 Erlenmeyer flasks (5 L) at 28 °C on a rotary shaker at 180 rpm for 16 d, which provided 13.9 g of the crude extract. Isolation and purification of the secondary metabolites were carried out mainly by column chromatography over silica gel, ODS, and sephadex LH-20.

**Figure 2:**
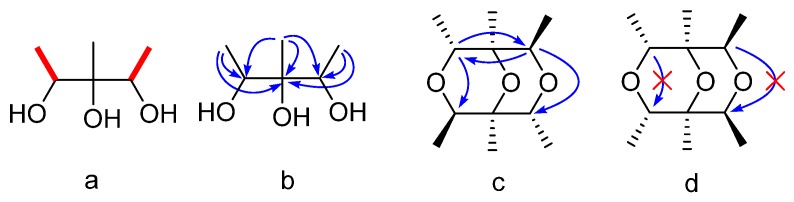
Key ^1^H-^1^H COSY (**a**) and HMBC (**b**, **c**, **d**) correlations of Compound **1**.

**Figure 3:**
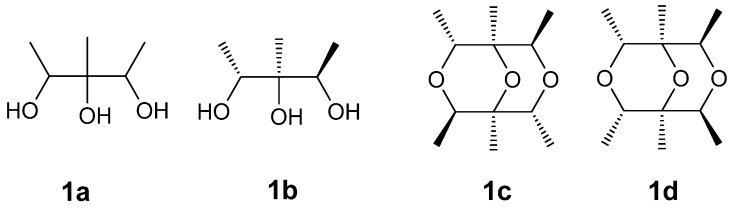
Chemical structure of **1a**, **1b**, **1c**, and **1d**.

Compound **1** was obtained as a colorless oil. Its molecular formula was determined as C_12_H_22_O_3_ on the basis of the prominent pseudomolecular peak at *m/z* 237.1464 ([M + Na]^+^) in the positive HRESIMS spectrum. The ^1^H-NMR spectrum exhibited two methyl doublets [δ_H_ 1.15 (3H, d, *J* = 6.3 Hz); 1.17 (3H, d, *J* = 6.3 Hz)], one methyl singlet (δ_H_ 1.03, 3H, s), and two oxygenated methines [δ_H_ 3.81 (1H, q, *J* = 6.4 Hz), 3.83 (1H, q, *J* = 6.4 Hz)]. These signals were supported by resonances in the ^13^C and Dept-135 NMR spectra, which exhibited six carbons comprising three methyls (δc 17.4 q, 17.6 q, and 17.8 q), two methines (δc 72.1 d, 72.2 d), and one oxygenated quaternary carbon at δc 76.2 s (Table 1). In the ^1^H-^1^H COSY spectrum, δ_H_ 1.15 and δ_H_ 1.17 were correlated to δ_H_ 3.81 and δ_H_ 3.83, respectively, indicating connections of δc 17.4 to δc 72.1 and δc 17.6 to δc 72.2 (a in Figure 2). Unfortunately, the poor quality of the HMBC correlations of **1** in CD_3_OD could not be used further to elucidate its structure because of the seriously overlapped signals. Alternatively, the deuterated DMSO was adopted and another new 1D and 2D NMR experiments were carried out. The significant correlations were found of δ_H_ 1.01 (3H, d, *J* = 6.4 Hz) to δ_C_ 69.2 d, δ_C_ 74.3 s; δ_H_ 0.89 s to δ_C_ 69.2 d, 74.3 s, and 70.3 d; δ_H_ 1.02 (3H, d, *J* = 6.4 Hz) to δ_C_ 74.3 s and δ_C_ 70.3 d (b in Figure 2). Therefore, the planar structure of **1** was deduced as 1,2,3-trimethylglycerol (**1a** in Figure 3). Since the chemical shift of δ_H_ 1.01/δ_H_ 3.68 and δ_C_ 17.5/δ_C_ 69.2 were slightly different from those of δ_H_ 1.02/δ_H_ 3.61 and δ_C_ 17.5/δ_C_ 70.3, the relative configuration of **1** could be determined as 1R*,2S*,3R*-trimethylglycerol (**1b** in Figure 3). Taking its molecular formula into consideration, **1** must be a symmetrical molecule constructed by **1b** through dehydroxylation reaction. This could also be confirmed by the absence of three hydroxyl signals of its ^1^H-NMR spectrum in DMSO-*d*_6_. Further HMBC correlations of δ_H_ 3.68 to δ_C_ 70.3 d, δ_H_ 3.61 to δ_C_ 69.2 d (*c* in Figure 2), and the missing correlations of δ_H_ 3.68 to δ_C_ 69.2, δ_H_ 3.61 to δ_C_ 70.3 (d in Figure 2) confirmed its structure must be **1c** instead of **1d** (Figure 3). On the basis of the above evidence, Compound **1** was determined as (1S*,2R*,4R*,5S*,6R*,8R*)-1,2,4,5,6,8-hexamethyl-3,7,9-trioxabicyclo[3.3.1]nonane.

By comparison of the NMR and MS spectroscopic data with those reported in the literature, 12 known compounds were then identified to be macrolactin A (**2**) [10], macrolactin F (**3**) [11], cyclo(d)-Pro-(d)-Leu (**4**) [12] cyclo-(Leu-Thr) (**5**) [13], thymidine (**6**) [14], 2′-*O*-methyluridine (**7**) [15], indol-3-carbaldehyde (**8**) [16], 2-(1*H*-indol-3-yl)ethan-1-ol (**9**) [17], 2-hydroxy phenyl acetic acid (**10**) [18], 4-(hydroxymethyl)phenol (**11**) [15], 2-(4-hydroxyphenyl)-ethanol (**12**) [19], and 2,3-butanediol (**13**) [20].

### 2.2. Anti-Allergic Activity

All 13 compounds were tested for their anti-allergic activities on IgE-mediated rat mast RBL-2H3 cells. Under the concentration of 20 μg/mL, nine compounds (**1**, **4**–**10**, and **13**) showed positive effects (Table 2). Compound **1** showed a moderate effect with an inhibition rate of 9.86%, while the positive control, loratadine, exhibited an inhibition rate of 37.41%. Compounds **4** and **8**, and loratadine, were subjected to further bioactive tests under eight concentrations of 1.0, 2.5, 5.0, 10.0, 20.0, 40.0, 60.0, and 80.0 μg/mL to yield IC_50_ values of 69.95, 57.12, and 35.01 μg/mL, respectively. The potent effect of **4** and **8** suggests their promising utilization as a functional food component or an active pharmaceutical ingredient for allergic patients.

## 3. Discussion

Food allergy is a potentially life-threatening condition affecting almost 10% of children, with increasing incidence in the last few decades [21]. As stated by the Codex Alimentarius Commission, food allergens include milk, eggs, fish, crustacean shellfish, peanuts, tree nuts, soybeans, and cereal sources [22]. Moreover, a food allergen-induced allergic reaction is usually accompanied by the promotion of serum-specific IgE. In fact, most food allergic reactions are serum IgE-mediated [23,24]. The IgE-mediated RBL-2H3 cell has been widely used as a mast cell model in food allergy studies [25]. After stimulation with antigen, mast cells release β-hexosaminidase, a marker of mast cell degranulation [26]. Presently, with the increasing prevalence of food allergies, anti-allergic testing is becoming increasingly commonplace [27]. However, there are no approved treatments other than avoidance [28,29]. Dietary intervention is a crucial component of food allergy management but can negatively impact nutrient intake and may influence growth and development [30]. Food allergy not only negatively impacts the health and quality of life of affected individuals but can also bring them significant economic burden [31]. Given the increasing prevalence and potential severity of food allergies, it is vital that therapeutic agents that either prevent sensitization to food antigens or suppress the allergic response after initiation are found. In this study, cyclo(d)-Pro-(d)-Leu (**4**) and indol-3-carbaldehyde (**8**) showed a significant anti-allergic effect on TM-specific IgE-mediated rat mast RBL-2H3 cells, indicating the potential application of these two compounds as anti-allergic medicines.

The genus of *Nesterenkonia* comprises Gram-positive nonspore-forming actinobacteria that may be either halotolerant or halophilic [32]. This was found for the first time by Stackebrandt et al. in 1995 [33]. Until now, the genus comprises 13 recognized species: *N. halobia*, *N. halotolerans*, *N. xinjiangensis*, *N. lutea*, *N. sandarakina*, *N. aethiopica*, *N. jeotgali*, *N. halophile*, *N. flava*, *N. alba*, *N. populi*, *N. alkaliphila*, and *N. aurantiaca*. Interestingly, this study was the first report on secondary metabolites from the genus of *Nesterenkonia*. Among these compounds, for the first time, **2**, **3**, **8**, and **9** were isolated from actinomycetes and **7**, **10**, and **13** from microorganisms.

## 4. Materials and Methods

### 4.1. General Experimental Procedures

Optical rotations were measured with an Anton Paar MCP500 polarimeter. NMR spectra were recorded on a Bruker Avance II 400 MHz spectrometer with TMS as an internal standard. HRESIMS were completed on a Waters Xevo G2 Q-TOF spectrometer. Materials for column chromatography were silica gel, Sephadex LH-20, and ODS.

### 4.2. Bacterial Material

The strain MCCC 1K00610 was isolated from a deep-sea water sample (−5302 m) of the Eastern Pacific Ocean in August 2013 by Prof. Xiao-Hua Zhang of the Ocean University of China. It was closely related to *Nesterenkonia flava* CCTCC AB 207010T EF680886 with a 16S rRNA gene sequence similarity value of 96.53%. A voucher strain of this actinomycete was deposited in the Marine Culture Collection of China (MCCC) under the accession number of MCCC 1K00610.

### 4.3. Cultivation and Extraction

The strain was cultured on solid-plates using 2216 E medium (Shanghai Yuanye Bio-Technology Co., Ltd.) for 5 d. Then, one plate of the actinomycetes was inoculated into a 1 L Erlenmeyer flask containing 200 mL of A14 broth (1.5% glycerin, 0.75% yeast extract powder, 0.75% tryptone, 1.5% sea salt, pH 7.2). After 48 h of incubation at 28 °C on a rotary shaker at 180 rpm, 200 mL of seed culture was transferred to a 5 L Erlenmeyer flask containing 2 L of A14 broth. Incubation was continued for 16 d at 28 °C, 180 rpm.

The whole culture medium (39.6 L from 18 5 L-Erlenmeyer flasks) was centrifuged to separate the supernatant and the mycelia cake. The supernatant was exhaustively extracted 3 times with ethyl acetate (EtOAc), and the mycelia cake was completely extracted with EtOAc followed by methanol (MeOH). After evaporation under vacuum, the extract of supernant (13.4 g) and the mycelia (0.5 g) were combined based on the result of thin layer chromatography (TLC) analysis to yield a brown gum (13.9 g).

### 4.4. Isolation and Purification

The crude extract (13.9 g) was subjected to medium pressure liquid chromatography (MPLC, 36 mm × 310 mm) on ODS using a gradient H_2_O–MeOH (15 mL/min) to yield five fractions (Fractions 1–5). Fraction 1 (700.1 mg) was column chromatographed (CC) over silica gel using petroleum ether (PE)–acetone (5:1) as an eluent to yield **13** (33.8 mg). Fraction 2 (829.0 mg) was CC on Sephadex LH-20 (3.0 × 180 cm, CHCl_3_–MeOH 1:1, 800 mL) to yield two subfractions Subfactions 2-1 and 2-2. Subfraction 2-1 was further CC over silica gel (CHCl_3_–acetone, 5:1) to yield **6** (1.3 mg) and **7** (8.2 mg), while Subfraction 2-2 was CC on silica gel (PE–acetone, 1:1) to provide **1** (5.6 mg) and **12** (7.6 mg). Fraction 3 (515.3 mg) was CC over silica gel (PE–acetone, 4:1) and Sephadex LH-20 (2.0 × 180 cm, MeOH, 500 mL) to yield **8** (11.0 mg) and **9** (2.1 mg). Similarly, Compounds **2** (6.7 mg) and **3** (5.4 mg) were obtained from Fraction 4 (397.6 mg). Fraction 5 (749.2 mg) was CC over silica gel (CHCl_3_–acetone, 5:1) and Sephadex LH-20 (3.0 × 180 cm, MeOH, 700 mL). Final purification by CC over silica gel afforded **10** (16.9 mg) together with **11** (2.1 mg) using CHCl_3_–acetone (5:1) and **4** (5.9 mg) together with **5** (2.3 mg) using PE–acetone (5:1).

*Nesterenkoniane* (**1**): Amorphous powder; ${\left[\alpha \right]}_{D}^{25}$ −1.57 (*c* 0.1, MeOH); ^1^H- and ^13^C-NMR data, see Table 1; HRESIMS (positive) [M + Na]^+^ *m/z* 237.1464, calcd. for C_12_H_22_O_3_Na^+^, 237.1461; [M + H]^+^ *m/z* 215.1644, calcd for C_12_H_23_O_3_^+^, 215.1642.

### 4.5. Anti-Allergic Bioassay

As previously reported [34], the release of β-hexosaminidase by RBL-2H3 cell was measured using IgE-mediated mast cell allergic reaction, using the sensitization of anti-DNP-IgE. The anti-allergic activity of tested compounds was determined for the efficiency of the cell degranulation inhibition rate.

Firstly, RBL-2H3 cells were digested by trypsin and seeded at 6 × 10^4^ cells per well in 96-well plates, and then incubated overnight with 1 μg/mL of anti-DNP-IgE (37 °C, 5% CO_2_) (Sigma, St. Louis, MO, USA). Secondly, the holes were washed 3 times with PBS, and the sample was dissolved by PBS and diluted with Tyrode’s buffer. IgE-sensitized RBL-2H3 cells were pre-treated with tested compounds (20 μg/mL) for 1 h and stimulated with 1 μg/mL DNP-BSA for 6 h in Tyrode’s buffer at 37 °C. The β-hexosaminidase activity of RBL-2H3 cells was measured with the 4-methylumbellife-ryl-*N*-acetyl-β-d-glucosaminide substrate. Thirdly, the cells were lysed with 0.1% Triton-X 100 prior to removing the supernatant for measurement of total activity. Supernatant or cell lysis liquid (25 μL) were put into 96-well plates respectively, and mixed with 4-methylumbellife-ryl-*N*-acetyl-β-d-glucosaminide (100 μL 1.2 mM) (Sigma, St. Louis, MO, USA), reacted for 30 min (37 °C). The β-hexosaminidase activity was quantified by measuring the fluorescence intensity of the hydrolyzed substrate in a fluorometer (Infinite M200PRO, Tecan, Zurich, Switzerland) using 360 nm excitation and 450 nm emission filters. The results were presented as the percentage of β-hexosaminidase activity measured in supernatant samples compared to the total levels in the corresponding cell lysates.

## 5. Conclusions

The systematic chemical investigation on deep-sea-derived actinomycete *Nesterenkonia flava* MCCC 1K00610 led to the isolation of one new and 12 known compounds. Cyclo(d)-Pro-(d)-Leu (**4**) and indol-3-carbaldehyde (**8**) exhibited significant anti-allergic activities with IC_50_ values of 69.95 and 57.12 μg/mL, respectively.

## Figures and Tables

**Table 1:** The ^1^H (400 MHz) and ^13^C (100 MHz) NMR spectroscopic data for **1**.

Position	^a^		^b^	
	δ_C_	δ_H_	δ_C_	δ_H_
1,5	76.2 s		74.3 s	
2,6	72.2 d	3.83 (1H, q, 6.4)	70.3 d	3.61 (1H, q, 6.4)
4,8	72.1 d	3.81 (1H, q, 6.4)	69.2 d	3.68 (1H, q, 6.0)
Me-1,5	17.8 q	1.03 (3H, s)	17.7 q	0.89 (3H, s)
Me-2,6	17.6 q	1.17 (3H, d, 6.3)	17.5 q	1.02 (3H, d, 6.4)
Me-4,8	17.4 q	1.15 (3H, d, 6.3)	17.5 q	1.01 (3H, d, 6.4)

^a^ Recorded in CD_3_OD; ^b^ Recorded in DMSO-*d*_6_.

**Table 2:** Anti-allergic effects of compounds from *Nesterenkonia flava* MCCC 1K00610.

Compounds	Inhibition Rate (%, 20 μg/mL)	IC_50_ (μg/mL)
**1**	9.86 ± 1.18	NT
**4**	16.99 ± 0.76	69.95 ± 2.34
**5**	7.28 ± 0.92	NT
**6**	7.83 ± 1.67	NT
**7**	8.73 ± 1.28	NT
**8**	16.38 ± 1.01	57.12 ± 7.67
**9**	12.96 ± 2.10	NT
**10**	6.02 ± 0.98	NT
**13**	9.96 ± 1.08	NT
OCs ^a^	>20.00	NT
Loratadine ^b^	37.41 ± 5.28	35.01 ± 0.48

Data were expressed as Means ± SD (*n* = 3). NT: not tested. ^a^ Other compounds, including **2**, **3**, **11**, and **12**; ^b^ Positive control.

## Supplementary Materials

The 1D and 2D NMR spectra in CD_3_OD and DMSO-*d*_6_ of **1** are available online at www.mdpi.com/1660-3397/15/3/71/s1 in Figures S1–S10.

## References

[1] Skropeta D., Wei L. (2014). Recent advances in deep-sea natural products. Nat. Prod. Rep..

[2] Zhang W., Yang C., Huang C., Zhang L., Zhang H., Zhang Q., Yuan C.S., Zhu Y., Zhang C. (2017). Pyrazolofluostatins A–C, pyrazole-fused benzo[a]fluorenes from South China Sea-derived *Micromonospora rosaria* SCSIO N160. Org. Lett..

[3] Zhang Y., Adnani N., Braun D.R., Ellis G.A., Barns K.J., Parker-Nance S., Guzei I.A., Bugni T.S. (2016). Micromonohalimanes A and B: Antibacterial halimane-type diterpenoids from a marine *Micromonospora* species. J. Nat. Prod..

[4] Nam S.J., Kauffman C.A., Jensen P.R., Moore C.E., Rheingold A.L., Fenical W. (2015). Actinobenzoquinoline and actinophenanthrolines A–C, unprecedented alkaloids from a aarine *Actinobacterium*. Org. Lett..

[5] Newman D.J., Cragg G.M. (2016). Natural products as sources of new drugs from 1981 to 2014. J. Nat. Prod..

[6] Kanani J., Banerjee M., Rajendran N. (2014). Drugs from marine microorganisms. Int. J. Pharm. Sci. Rev. Res..

[7] Janardhan A., Kumar A.P., Viswanath B., Saigopal D.V., Narasimha G. (2014). Production of bioactive compounds by actinomycetes and their antioxidant properties. Biotechnol. Res. Int..

[8] Jensen P.R., Moore B.S., Fenical W. (2015). The marine actinomycete genus Salinispora: A model organism for secondary metabolite discovery. Nat. Prod. Rep..

[9] Abdelmohsen U.R., Bayer K., Hentschel U. (2014). Diversity, abundance and natural products of marine sponge-associated actinomycetes. Nat. Prod. Rep..

[10] He S., Wang H., Yan X., Zhu P., Chen J., Yang R. (2013). Preparative isolation and purification of macrolactin antibiotics from marine bacterium *Bacillus amyloliquefaciens* using high-speed counter-current chromatography in stepwise elution mode. J. Chromatogr. A.

[11] Tomokazu N., Kyoko A., Miho S., Miyuki N., Hiroshi S. (2001). Novel macrolactins as antibiotic lactones from a marine bacterium. J. Antibiot..

[12] Fdhila F., Vazquez V., Sanchez J.L., Riguera R. (2003). dd-Diketopiperazines: Antibiotics active against Vibrio anguillarum isolated from marine bacteria associated with cultures of *Pecten maximus*. J. Nat. Prod..

[13] Tan N.H., Wang S.M., Yang Y.B., He M. (2003). Cyclodipeptides of Panax notoginseng and Lactams of Panas ginseng. Yunnan Zhiwu Yanjiu.

[14] Youssef D.T.A., Badr J.M., Shaala L.A., Mohamed G.A., Bamanie F.H. (2015). Ehrenasterol and biemnic acid; new bioactive compounds from the Red Sea sponge *Biemna ehrenbergi*. Phytochem. Lett..

[15] Du W.P., Xu P., Liu B., Xu X.H., Lai X.W., Li B. (2015). Chemical constituents from shoots of *Phyllostachys edulis* (I). Zhongcaoyao.

[16] Asiri I.A.M., Badr J.M., Youssef D.T.A. (2015). Penicillivinacine, antimigratory diketopiperazine alkaloid from the marine-derived fungus *Penicillium vinaceum*. Phytochem. Lett..

[17] Böhlendorf B., Bedorf N., Jansen R., Wolfram T.K., Höfle G., Forche E., Gerth K., Irschik H., Kunze B., Reichenbach H. (1996). Indole and quinoline derivatives as metabolites of tryptophan in *Myxobacteria*. Eur. J. Org. Chem..

[18] Gutierrezlugo M.T., Woldemichael G.M., Singh M.P., Suarez P.A., Maiese W.M., Montenegro G., Timmermann B.N. (2005). Isolation of three new naturally occurring compounds from the culture of *Micromonospora* sp. P1068. Nat. Prod. Res..

[19] Lou H.X., Yuan H.Q., Yamazaki Y., Sasaki T., Oka S. (2001). Alkaloids and flavonoids from peanut skins. Planta Med..

[20] Uemura Y., Sugimoto S., Matsunami K., Otsuka H., Takeda Y., Kawahata M., Yamaguchi K. (2013). Microtropins A–I: 6′-*O*-(2″*S*,3″*R*)-2″-Ethyl-2″,3″-dihydroxybutyrates of aliphatic alcohol β-d-glucopyranosides from the branches of *Microtropis japonica*. Phytochemistry.

[21] Bergmann M.M., Eigenmann P.A. (2015). Food allergy in childhood (infancy to school age). Chem. Immunol. Allergy.

[22] Taylor S.L., Baumert J.L. (2015). Worldwide food allergy labeling and detection of allergens in processed foods. Chem. Immunol. Allergy.

[23] Chen C.Y., Lee J.B., Liu B., Ohta S., Wang P.Y., Kartashov A.V., Mugge L., Abonia J.P., Barski A., Izuhara K. (2015). Induction of interleukin-9-producing mucosal mast cells promotes susceptibility to IgE-mediated experimental food allergy. Immunity.

[24] Moriyama T. (2015). Diversity of food allergy. J. Nutr. Sci. Vitaminol..

[25] Passante E., Ehrhardt C., Sheridan H., Frankish N. (2009). RBL-2H3 cells are an imprecise model for mast cell mediator release. Inflamm. Res..

[26] Gilfillan A.M., Tkaczyk C. (2006). Integrated signalling pathways for mast-cell activation. Nat. Rev. Immunol..

[27] Chokshi N.Y., Sicherer S.H. (2016). Interpreting IgE sensitization tests in food allergy. Expert. Rev. Clin. Immunol..

[28] Burbank A.J., Burks W. (2015). Food specific oral immunotherapy: A potential treatment for food allergy. Expert Rev. Gastroenterol. Hepatol..

[29] Carrard A., Rizzuti D., Sokollik C. (2015). Update on food allergy. Allergy.

[30] Nowak W.A., Groetch M. (2015). Nutritional aspects and diets in food allergy. Chem. Immunol. Allergy.

[31] Sharma H.P., Herbert L.J. (2015). Food allergy: Psychosocial impact and public policy implications. Chem. Immunol. Allergy.

[32] Govender L., Naidoo L., Setati M.E. (2013). *Nesterenkonia suensis* sp. nov., a haloalkaliphilic actinobacterium isolated from a salt pan. Int. J. Syst. Evol. Microbiol..

[33] Stackebrandt E., Koch C., Gvozdiak O., Schumann P. (1995). Taxonomic dissection of the genus Micrococcus: *Kocuria gen*. nov., *Nesterenkonia gen*. nov., *Kytococcus gen*. nov., *Dermacoccus gen*. nov., and Micrococcus Cohn 1872 gen. emend. Int. J. Syst. Bacteriol..

[34] Liu Q., Wang Y., Cao M., Pan T., Yang Y., Mao H., Sun L., Liu G. (2015). Anti-allergic activity of R-phycocyanin from *Porphyra haitanensis* in antigen-sensitized mice and mast cells. Int. Immunopharmacol..

