# Supplementary material for: Anti-Allergic Compounds from the Deep-Sea-Derived Actinomycete Nesterenkonia flava MCCC 1K00610
Source: Mar Drugs. 2017 Mar 14;15(3):71. doi: 10.3390/md15030071 (PMC5367028; doi:10.3390/md15030071)
Supplement: Supplementary file 1 [file marinedrugs-15-00071-s001.pdf]

Figure S1. ^{1}H NMR spectrum of compound 1 in CD_3OD

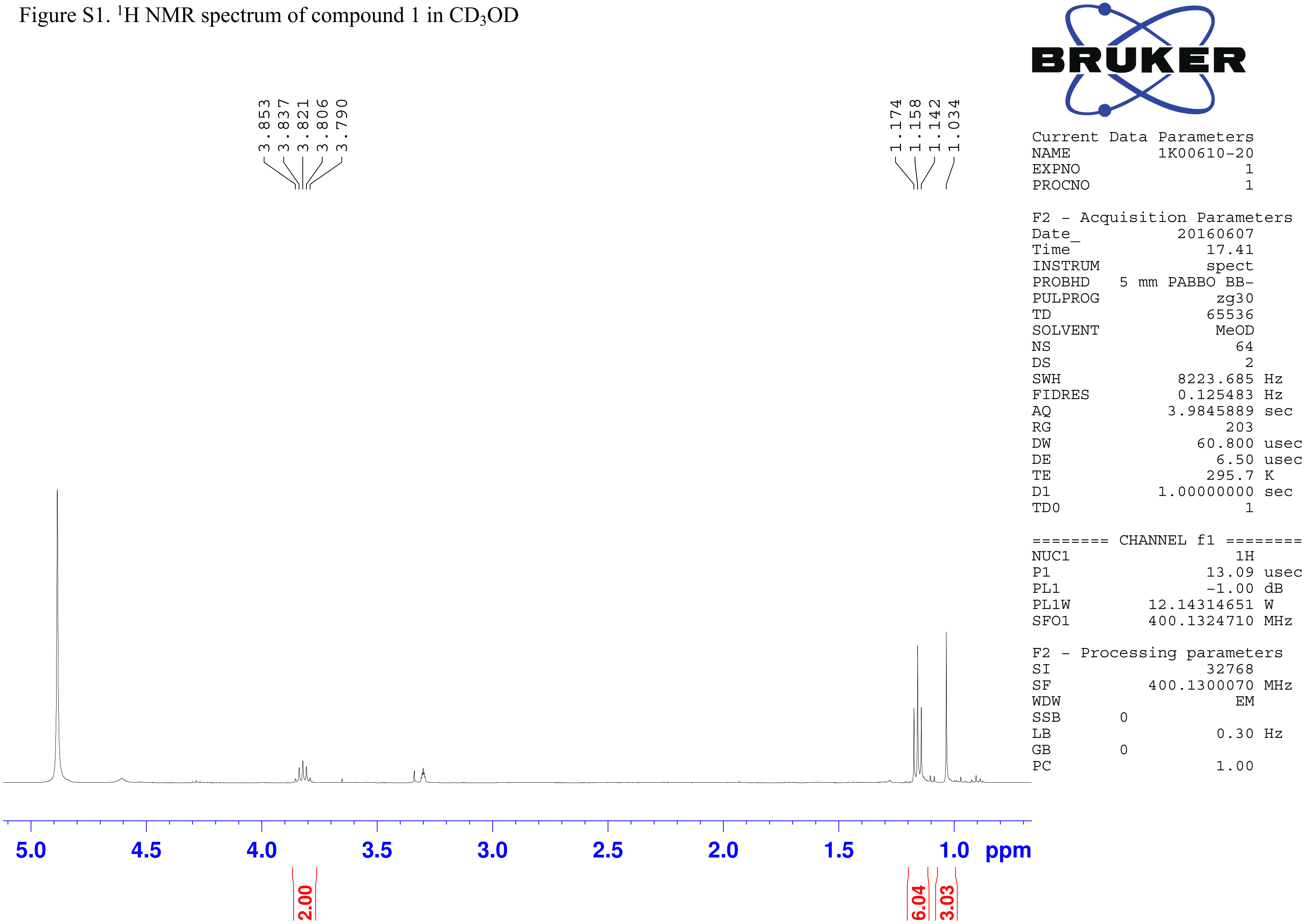

Figure S2. ^{13}C NMR spectrum of compound 1 in CD_3OD

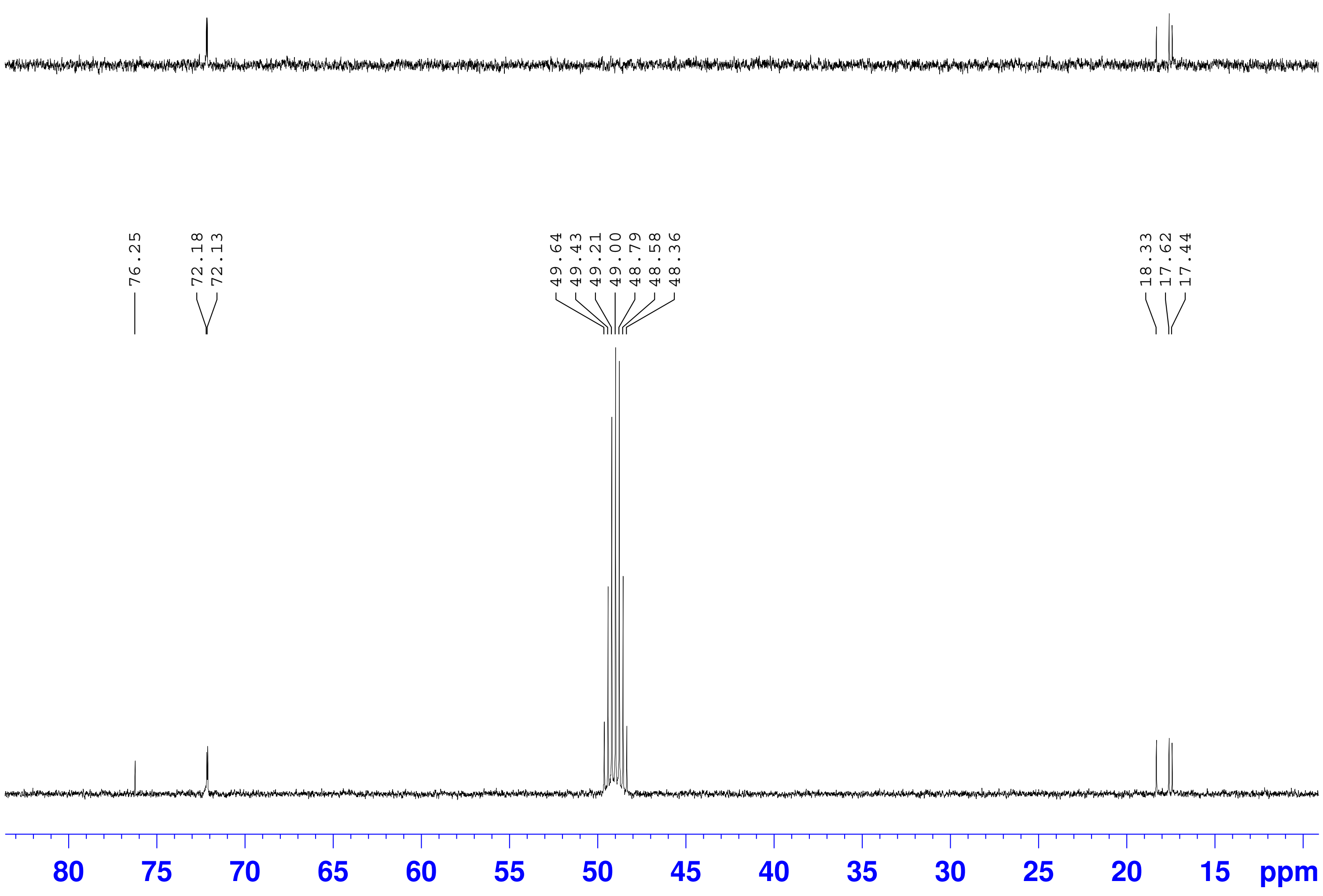

Figure S3. HSQC spectrum of compound 1 in CD_3OD

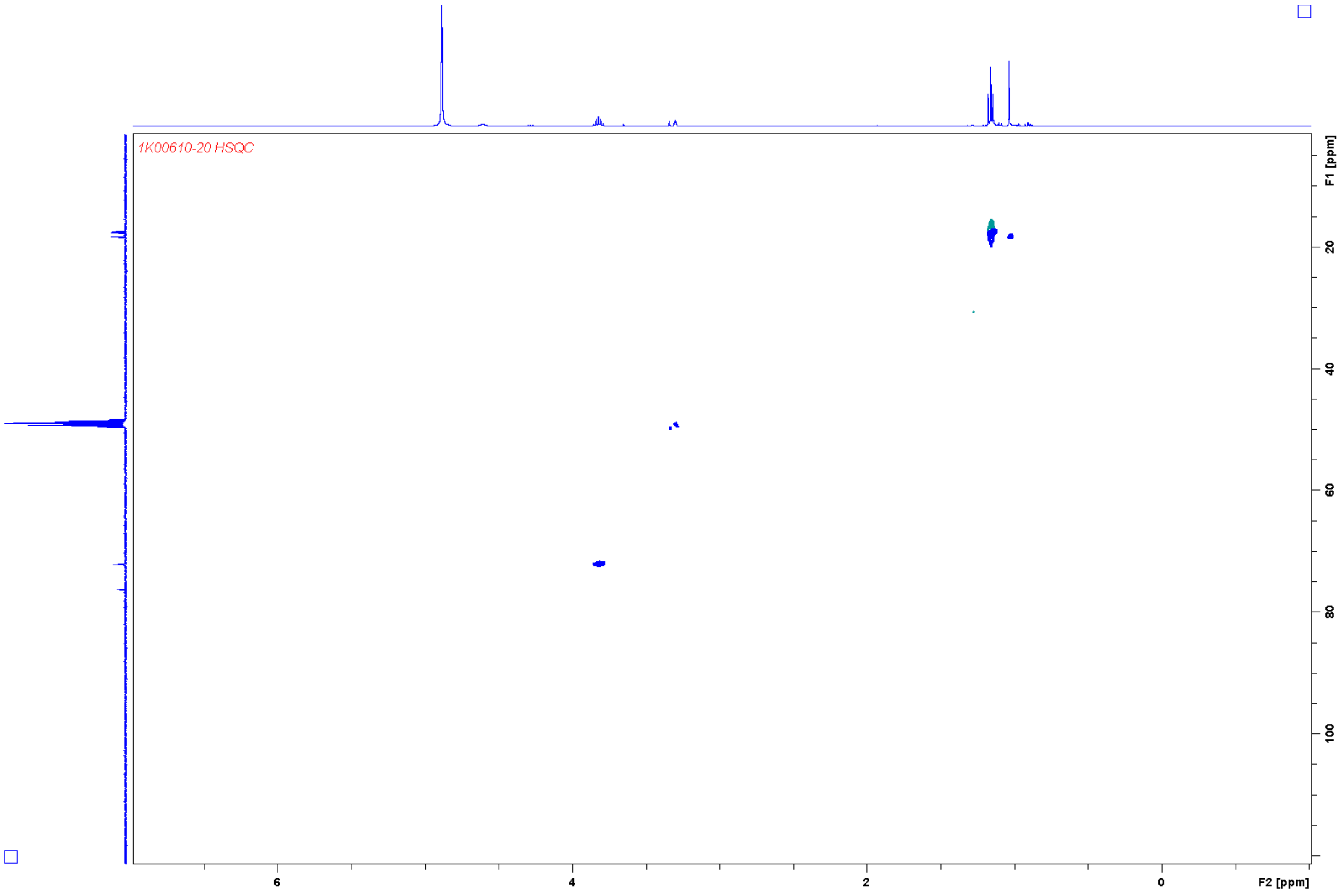


```
Current Data Parameters
NAME           1K00610-20
EXPNO                   4
PROCNO                  1

F2 - Acquisition Parameters
Date_            20160610
Time                19.33
INSTRUM             spect
PROBHD   5 mm PABBO BB-
PULPROG        hsqcedetgp
TD                   1024
SOLVENT              MeOD
NS                      8
DS                     32
SWH              3201.024 Hz
FIDRES           3.126000 Hz
AQ              0.1599488 sec
RG                    203
DW                156.200 usec
DE                   6.50 usec
TE                  295.6 K
CNST2         145.0000000
D0             0.00000300 sec
D1             1.50000000 sec
D4             0.00172414 sec
D11            0.03000000 sec
D13            0.00000400 sec
D16            0.00020000 sec
D21            0.00345000 sec
IN0            0.00004140 sec
ZGOPTNS

======== CHANNEL f1 ========
NUC1                   1H
P1                  13.09 usec
P2                  26.18 usec
P28                  1.00 usec
PL1                 -1.00 dB
PL1W          12.14314651 W
SFO1          400.1312004 MHz

======== CHANNEL f2 ========
CPDPRG[2             garp
NUC2                  13C
P3                  12.37 usec
P4                  24.74 usec
PCPD2               75.00 usec
PL2                  1.00 dB
PL12                16.65 dB
PL2W          28.13319778 W
PL12W          0.76598305 W
SFO2          100.6188058 MHz

====== GRADIENT CHANNEL =====
GPNAM[1]         SINE.100
GPNAM[2]         SINE.100
GPZ1                80.00 %
GPZ2                20.10 %
P16               1000.00 usec

F1 - Acquisition parameters
TD                    256
SFO1             100.6188 MHz
FIDRES          47.165066 Hz
SW                120.000 ppm
FnMODE       Echo-Antiecho

F2 - Processing parameters
SI                   1024
SF            400.1300070 MHz
WDW                 QSINE
SSB                     2
LB         0 Hz
GB         0
PC                   1.40

F1 - Processing parameters
SI                   1024
MC2          echo-antiecho
SF            100.6126271 MHz
WDW                 QSINE
SSB                     2
LB         0 Hz
GB         0
```

Figure S4. ^{1}H–^{1}H COSY spectrum of compound 1 in CD_3OD

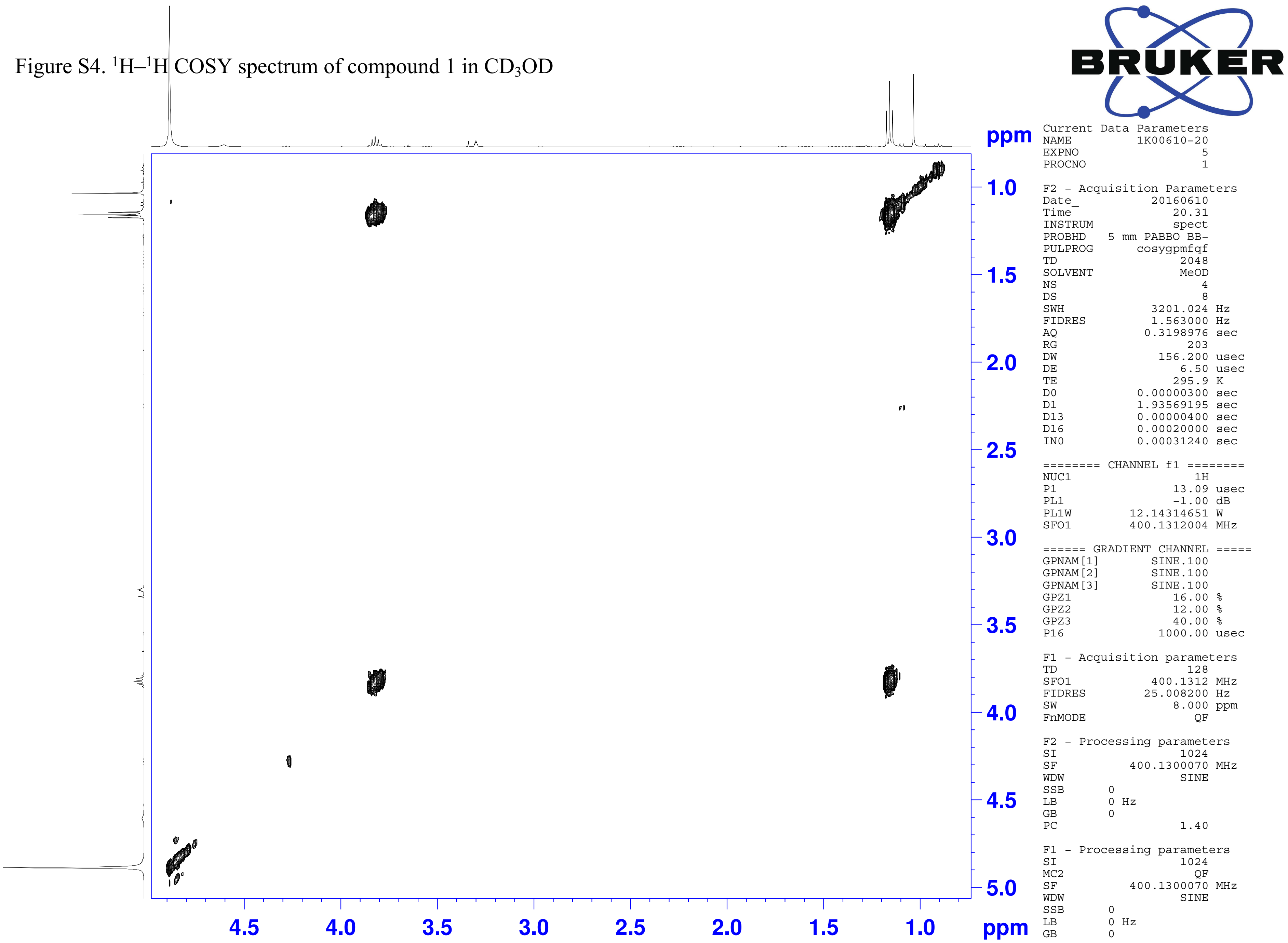

Figure S5. HMBC spectrum of compound 1 in CD_3OD

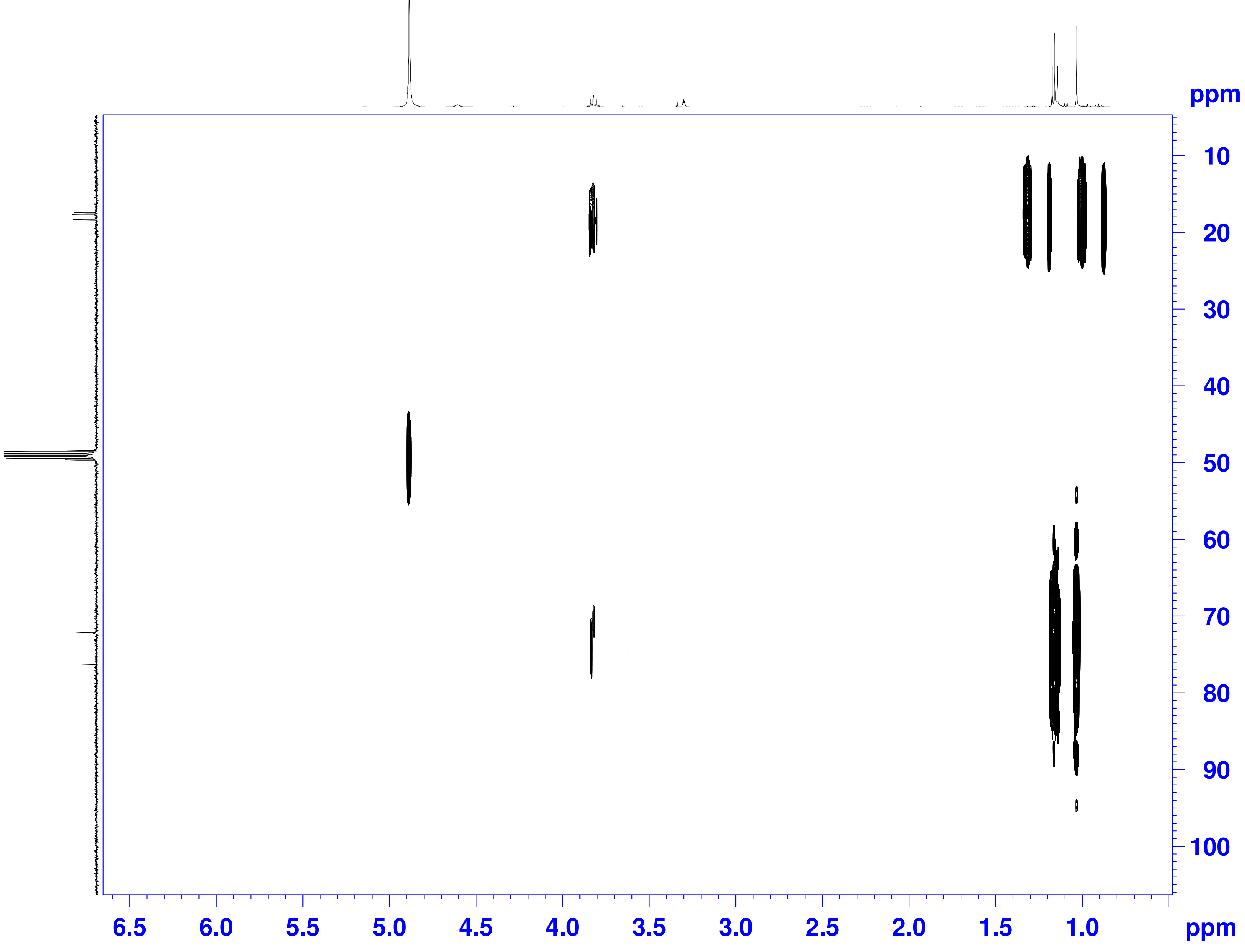


```
Current Data Parameters
NAME           1K00610-20
EXPNO                   6
PROCNO                  1

F2 - Acquisition Parameters
Date_            20160610
Time                20.54
INSTRUM             spect
PROBHD    5 mm PABBO BB-
PULPROG        hmbcgpndqf
TD                   4096
SOLVENT              MeOD
NS                     64
DS                     16
SWH              3201.024 Hz
FIDRES           0.781500 Hz
AQ              0.6397952 sec
RG                    203
DW                156.200 usec
DE                   6.50 usec
TE                  295.7 K
CNST13          8.0000000
D0             0.00000300 sec
D1             1.37220395 sec
D6             0.06250000 sec
D16            0.00020000 sec
IN0            0.00004140 sec

======== CHANNEL f1 ========
NUC1                   1H
P1                  13.09 usec
P2                  26.18 usec
PL1                 -1.00 dB
PL1W          12.14314651 W
SFO1          400.1312004 MHz

======== CHANNEL f2 ========
NUC2                  13C
P3                  12.37 usec
PL2                  1.00 dB
PL2W          28.13319778 W
SFO2          100.6188058 MHz

====== GRADIENT CHANNEL =====
GPNAM[1]         SINE.100
GPNAM[2]         SINE.100
GPNAM[3]         SINE.100
GPZ1                50.00 %
GPZ2                30.00 %
GPZ3                40.10 %
P16               1000.00 usec

F1 - Acquisition parameters
TD                     11
SFO1             100.6188 MHz
FIDRES        1097.659668 Hz
SW                120.000 ppm
FnMODE                 QF

F2 - Processing parameters
SI                   1024
SF            400.1300070 MHz
WDW                  SINE
SSB        0
LB         0 Hz
GB         0
PC                   1.40

F1 - Processing parameters
SI                   1024
MC2                    QF
SF            100.6126271 MHz
WDW                  SINE
SSB        0
LB         0 Hz
GB         0
```

Figure S6. ^{1}H NMR spectrum of compound 1 in DMSO-d_6

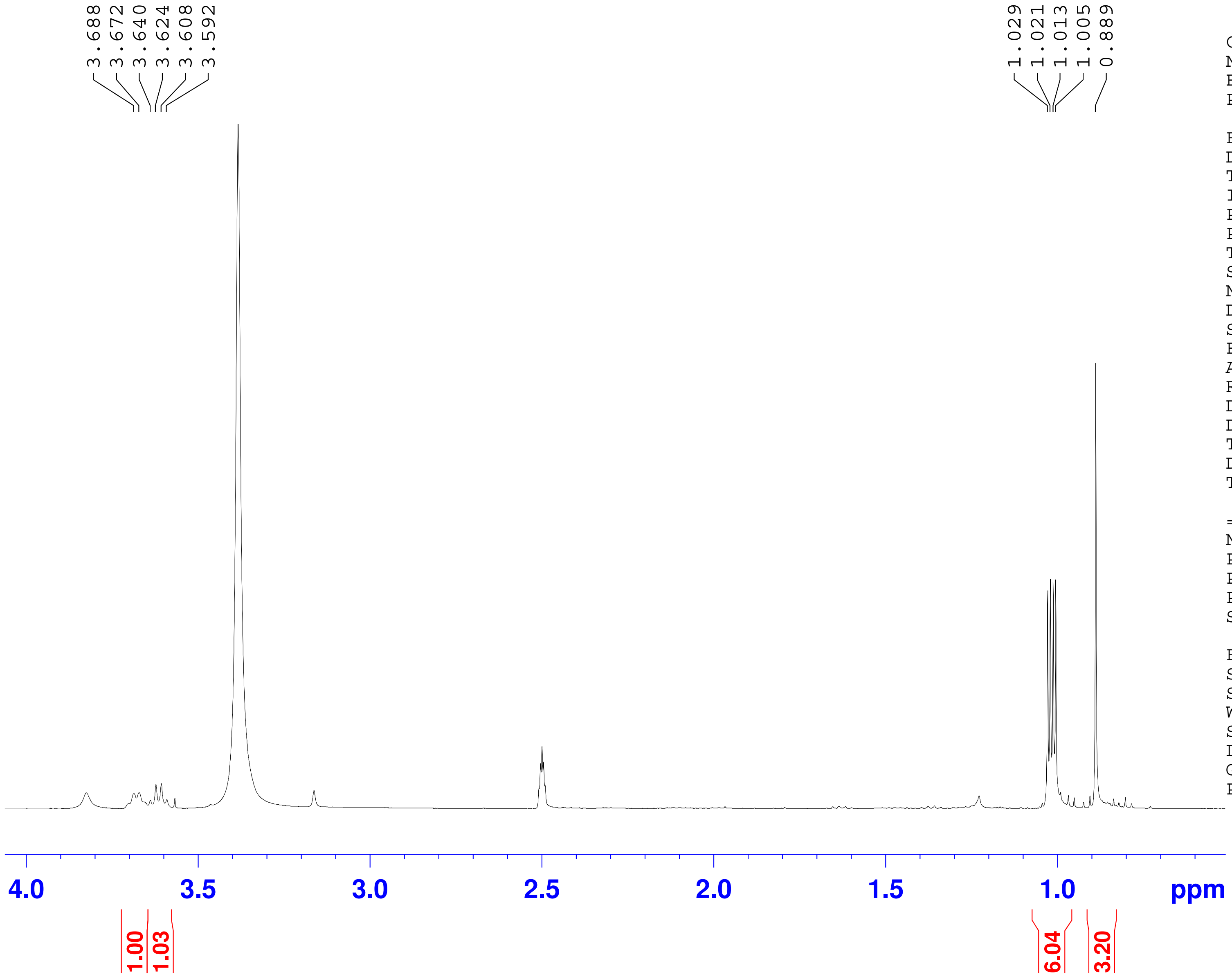


```
Current Data Parameters
NAME      1K00610-20 DMSO
EXPNO                   1
PROCNO                  1

F2 - Acquisition Parameters
Date_            20160815
Time                18.10
INSTRUM             spect
PROBHD     5 mm PABBO BB-
PULPROG              zg30
TD                  65536
SOLVENT              DMSO
NS                    128
DS                      2
SWH              8223.685 Hz
FIDRES           0.125483 Hz
AQ              3.9845889 sec
RG                    203
DW                 60.800 usec
DE                   6.50 usec
TE                  295.8 K
D1             1.00000000 sec
TD0                     1

======== CHANNEL f1 ========
NUC1                   1H
P1                  13.09 usec
PL1                 -1.00 dB
PL1W          12.14314651 W
SFO1          400.1324710 MHz

F2 - Processing parameters
SI                  32768
SF            400.1299972 MHz
WDW                    EM
SSB      0
LB                   0.30 Hz
GB       0
PC                   1.00
```

Figure S7. ^{13}C NMR spectrum of compound 1 in DMSO-d_6

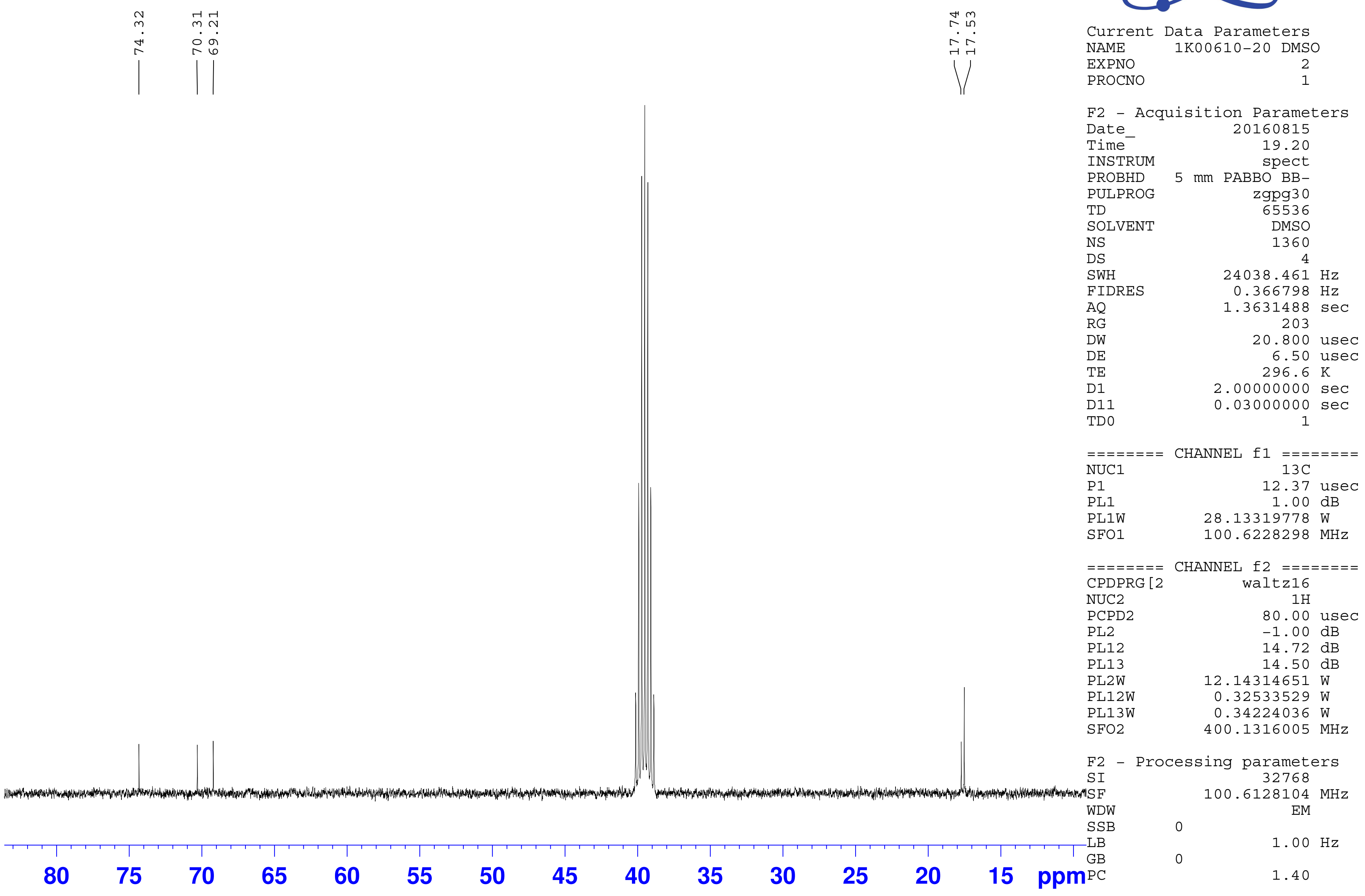

Figure S8. HSQC spectrum of compound 1 in DMSO-d_6

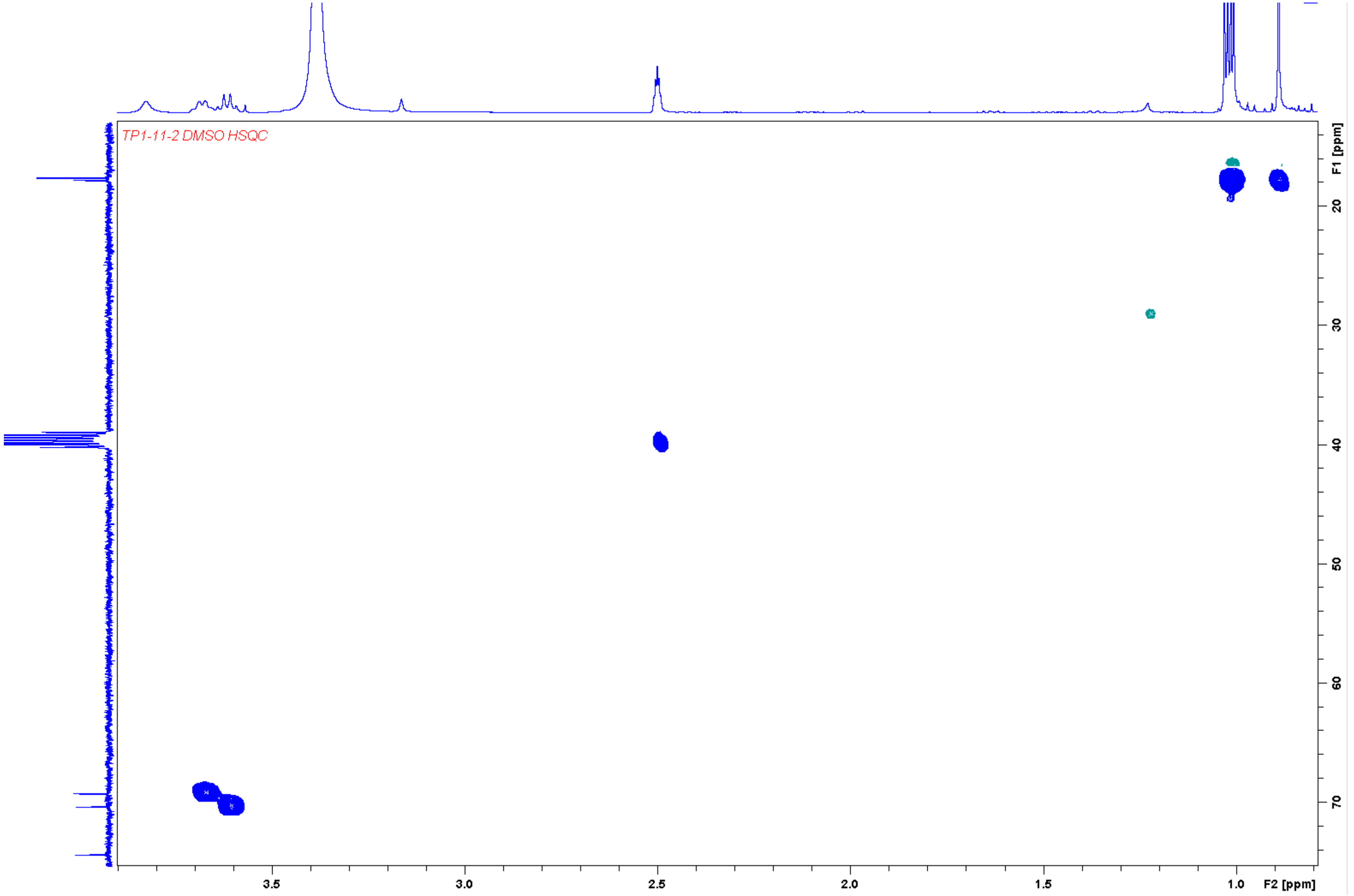


```
Current Data Parameters
NAME      1K00610-20 DMSO
EXPNO                   3
PROCNO                  1

F2 - Acquisition Parameters
Date_            20160815
Time                19.30
INSTRUM             spect
PROBHD    5 mm PABBO BB-
PULPROG       hsqcedetgp
TD                   1024
SOLVENT              DMSO
NS                     16
DS                     32
SWH              4401.409 Hz
FIDRES           4.298251 Hz
AQ              0.1163264 sec
RG                    203
DW                113.600 usec
DE                   6.50 usec
TE                  296.2 K
CNST2         145.0000000
D0             0.00000300 sec
D1             1.50000000 sec
D4             0.00172414 sec
D11            0.03000000 sec
D13            0.00000400 sec
D16            0.00020000 sec
D21            0.00345000 sec
IN0            0.00004140 sec
ZGOPTNS

======== CHANNEL f1 ========
NUC1                   1H
P1                  13.09 usec
P2                  26.18 usec
P28                  1.00 usec
PL1                 -1.00 dB
PL1W          12.14314651 W
SFO1          400.1318006 MHz

======== CHANNEL f2 ========
CPDPRG[2             garp
NUC2                  13C
P3                  12.37 usec
P4                  24.74 usec
PCPD2               75.00 usec
PL2                  1.00 dB
PL12                16.65 dB
PL2W          28.13319778 W
PL12W          0.76598305 W
SFO2          100.6188058 MHz

====== GRADIENT CHANNEL =====
GPNAM[1]         SINE.100
GPNAM[2]         SINE.100
GPZ1                80.00 %
GPZ2                20.10 %
P16               1000.00 usec

F1 - Acquisition parameters
TD                    112
SFO1             100.6188 MHz
FIDRES         107.805862 Hz
SW                120.000 ppm
FnMODE       Echo-Antiecho

F2 - Processing parameters
SI                   1024
SF            400.1299972 MHz
WDW                 QSINE
SSB                     2
LB        0 Hz
GB        0
PC                   1.40

F1 - Processing parameters
SI                   1024
MC2          echo-antiecho
SF            100.6128104 MHz
WDW                 QSINE
SSB                     2
LB        0 Hz
GB        0
```

Figure S9. ^{1}H–^{1}H COSY spectrum of compound **1** in DMSO-d_6

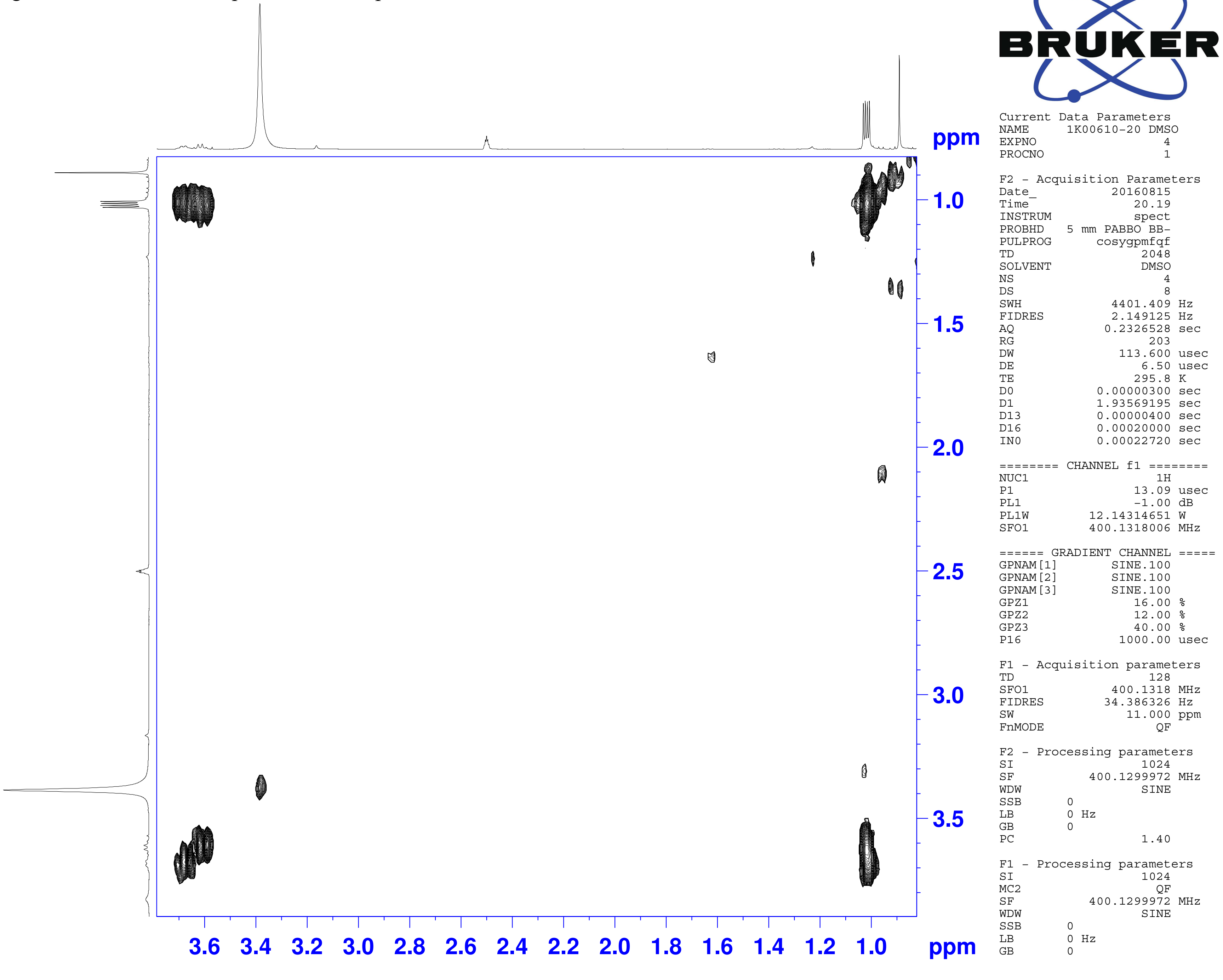

Figure S10. HMBC spectrum of compound 1 in DMSO-d_6

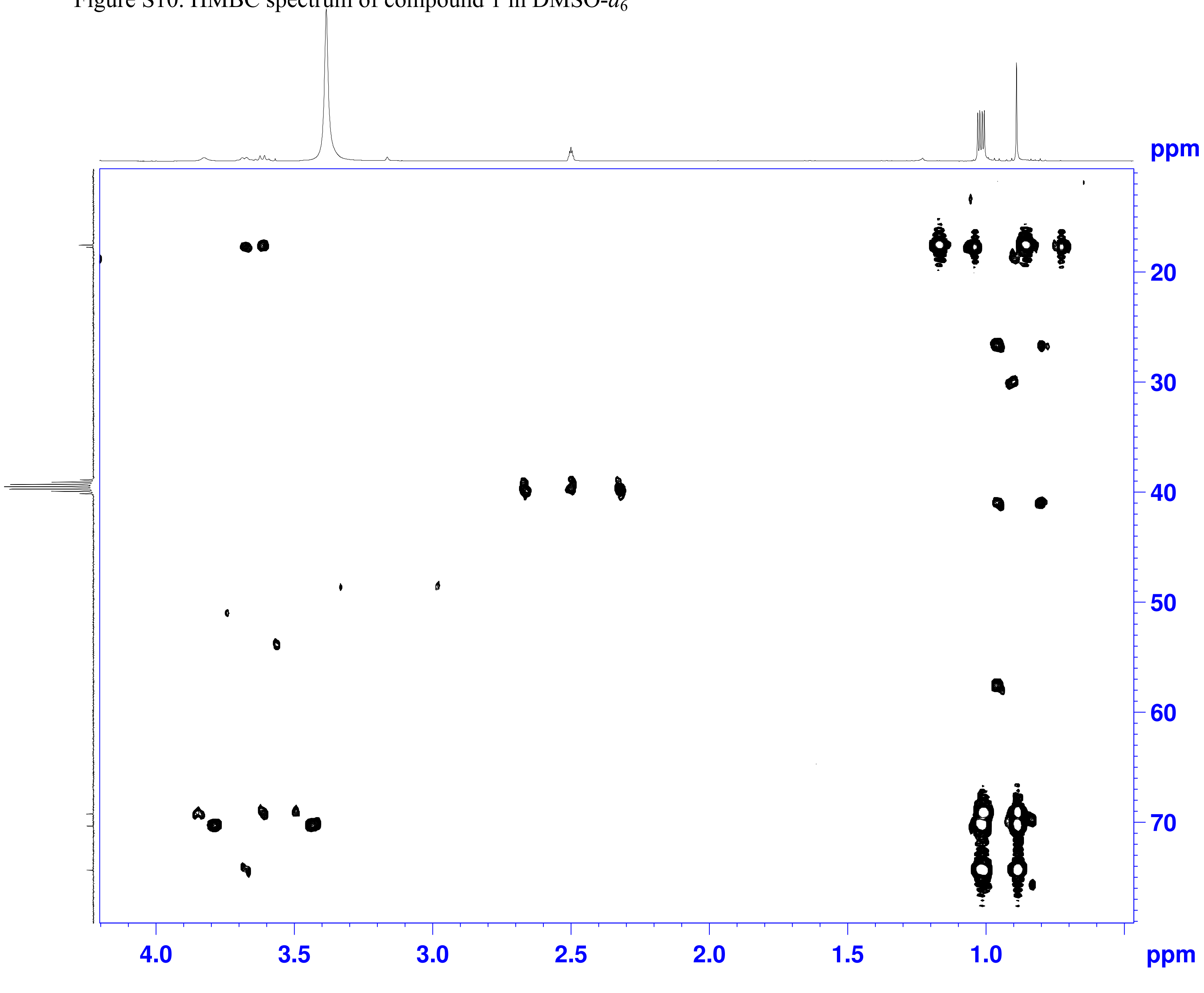


```
Current Data Parameters
NAME      1K00610-20 DMSO
EXPNO                   7
PROCNO                  1

F2 - Acquisition Parameters
Date_            20160815
Time                22.29
INSTRUM             spect
PROBHD    5 mm PABBO BB-
PULPROG        hmbcgpndqf
TD                   4096
SOLVENT              DMSO
NS                    128
DS                     16
SWH              4401.409 Hz
FIDRES           1.074563 Hz
AQ              0.4653056 sec
RG                    203
DW                113.600 usec
DE                   6.50 usec
TE                  295.5 K
CNST13          8.0000000
D0             0.00000300 sec
D1             1.37220395 sec
D6             0.06250000 sec
D16            0.00020000 sec
IN0            0.00004140 sec

======== CHANNEL f1 ========
NUC1                   1H
P1                  13.09 usec
P2                  26.18 usec
PL1                 -1.00 dB
PL1W          12.14314651 W
SFO1          400.1318006 MHz

======== CHANNEL f2 ========
NUC2                  13C
P3                  12.37 usec
PL2                  1.00 dB
PL2W          28.13319778 W
SFO2          100.6188058 MHz

====== GRADIENT CHANNEL =====
GPNAM[1]         SINE.100
GPNAM[2]         SINE.100
GPNAM[3]         SINE.100
GPZ1                50.00 %
GPZ2                30.00 %
GPZ3                40.10 %
P16               1000.00 usec

F1 - Acquisition parameters
TD                    128
SFO1             100.6188 MHz
FIDRES          94.330132 Hz
SW                120.000 ppm
FnMODE                 QF

F2 - Processing parameters
SI                   1024
SF            400.1299972 MHz
WDW                  SINE
SSB      0
LB       0 Hz
GB       0
PC                   1.40

F1 - Processing parameters
SI                   1024
MC2                    QF
SF            100.6128104 MHz
WDW                  SINE
SSB      0
LB       0 Hz
GB       0
```
